# The Thermomechanical, Functional and Biocompatibility Properties of a Au–Pt–Ge Alloy for PFM Dental Restorations

**DOI:** 10.3390/ma17225491

**Published:** 2024-11-10

**Authors:** Peter Majerič, Minja Miličić Lazić, Dijana Mitić, Marko Lazić, Ema Krdžović Lazić, Gyöngyi Vastag, Ivan Anžel, Vojkan Lazić, Rebeka Rudolf

**Affiliations:** 1 Faculty of Mechanical Engineering, University of Maribor, 2000 Maribor, Slovenia; peter.majeric@um.si (P.M.); ivan.anzel@um.si (I.A.); 2 ZlatarnaCelje d.o.o., 3000 Celje, Slovenia; 3 School of Dental Medicine, University of Belgrade, 11000 Belgrade, Serbia; minja.milicic@stomf.bg.ac.rs (M.M.L.); dijana.trisic@stomf.bg.ac.rs (D.M.); marko.lazic@stomf.bg.ac.rs (M.L.); ema.krdzovic@stomf.bg.ac.rs (E.K.L.); vojkan.lazic@stomf.bg.ac.rs (V.L.); 4 Faculty of Sciences, University of Novi Sad, 21000 Novi Sad, Serbia; djendji.vastag@dh.uns.ac.rs; 5 Pomurje Science and Innovation Centre, 9000 Murska Sobota, Slovenia

**Keywords:** noble metal dental alloys, metal–ceramic alloys, materials testing, biocompatibility testing

## Abstract

A high-noble Au–Pt–Ge porcelain-fused-to-metal (PFM) dental alloy without the known adverse metallic elements and with the addition of germanium (Ge) was produced as a more cost-effective alternative to other precious alloying metals, with investigations for determining the functionality and clinical use of this alloy. The thermomechanical, biocompatibility, durability, workability and economic characteristics of the produced dental alloy were investigated. These properties were investigated with in vitro biocompatibility testing on human gingival fibroblasts (HGFs); static immersion testing for metal ion release; DSC analysis; hardness, tensile testing, density and coefficient of thermal expansion (CTE) measurements; metallographic and SEM/EDX microstructure investigations; and finally with the production of a test PFM dental bridge. The results of the thermomechanical testing showed alloy properties suitable for dental restorations and clinical use, with somewhat lower mechanical properties, making the alloy not suitable for extensive multiunit fixed restorations. The microstructure investigations showed segregations of Ge in the homogeneous alloy matrix, which reduce the alloy’s mechanical properties. The produced PFM dental bridge showed excellent workability of the alloy in a dental laboratory setting, as well as a high standard of the final dental restoration. The ion release was negligible, well below any harmful quantities, while the cell viability examination showed significantly higher viability ratings on polished alloy samples as compared to as-cast samples. The results showed that a dental substructure in direct contact with oral tissue and fluids should be highly polished. The performed investigations showed that the produced PFM dental alloy is suitable for clinical use in producing high-quality dental restorations with high biocompatibility for patients prone to metal allergies.

## 1. Introduction

Considering the historical development and evolution of dental alloys, the first materials used in dentistry were gold-based alloys. Gold is considered a superior metallic biomaterial due to its exceptional resistance to corrosion, which makes it ideal for noble metal applications in biologically sensitive environments. Its unique colour distinguishes gold from most other metals, except copper and caesium. In developing alloys for fixed dental prostheses, gold is also employed extensively because of its high electrical conductivity (conductive metals resist tarnishing and corrosion, ensuring the longevity and stability of dental restorations) and strong bonding with porcelain [1]. These characteristics make it an ideal candidate for producing inlays, crowns and substructures for applications in dentistry. High-noble alloys based on gold and platinum have been favoured in dentistry for their proven long-term use and clinical reliability.

Regarding economic pressure and the increasing cost of noble metals, the exploration of more cost-effective alternatives is making base alloys like CoCr attractive options. Besides lower prices, CoCr alloys provide satisfactory mechanical performance in long-term clinical use, with durability and wear resistance comparable to those of noble alloys [2,3]. However, there has been an increase in reports from clinical studies highlighting both biological and technological complications related to base metal alloys. In both in vitro and in vivo investigations there, is evidence that dental restorations release metal ions, due primarily to corrosion [4]. Due to the challenging conditions in oral environments, chemical- and microorganism-induced corrosion are most likely to develop [5]. Metal ions can be released locally and systemically, potentially contributing to the development of oral and systemic health issues. The type of alloy and various corrosion factors significantly influence the types and amounts of cations released. Dental prostheses deal with oral environment-dependent factors for extended periods, facing corrosive impacts such as saliva exposure, temperature and pH fluctuations, among others. The release of ions from metals in saliva can initiate oxidative reactions, leading to discolouration and the deposition of metal in the adjacent tissues. Health concerns are particularly significant for alloys containing nickel as an alloying element [6,7]. Recently, germanium (Ge) has been identified as a promising alternative alloying element [8]. Previous research has revealed that adding germanium (Ge) to alloys results in reduced hardness, improved flowability and less shrinkage during casting while also offering a more cost-effective alternative to other precious metals [8,9].

Porcelain-fused-to-metal (PFM) fixed restorations often require subgingival margin preparation, which calls for a thorough evaluation of the host’s biological response to specific metallic materials [10]. To ensure these biomaterials remain within the physiological tolerance levels, it is important to examine the direct effects of different alloys on the host tissue, not only in their as-cast form but also after thermomechanical and degassing treatments. For instance, the presence of oxide alloying elements formed during these processes may modulate or alter the host response.

There is little knowledge about the cytotoxic effect of the degassing process on noble porcelain-fused-to-metal restorations.

Besides biological concerns, technological challenges in dental restorations, such as the higher incidence of porcelain veneer chipping observed in base alloy substructures (18–25%) compared to noble alloys (4–10%), have been reported extensively [11]. The main factors contributing to this discrepancy are thermal expansion compatibility and the characteristics of oxide layer formation [12]. Noble alloys generally have a thermal expansion rate that matches porcelain closely, reducing thermal stress during cooling and firing and thus minimising chipping. In contrast, base alloys often exhibit a significant mismatch in thermal expansion, leading to increased stress and a higher incidence of chipping [13]. Additionally, base alloys form a thick, irregular oxide layer that can weaken the bond with porcelain, further contributing to the higher chipping rates. In comparison, noble alloys form a stable oxide layer that strengthens the bond, reducing the risk of chipping.

Moreover, one study, which analysed the discrepancy in chemical composition, revealed that base metal alloys differed more than noble alloys. The number of compositional discrepancies for gold alloys was 8% (1 of 13), and that for base alloys was 31% (4 of 13) [14]. Unlike base alloys, noble alloys are less reactive with the environment, maintaining their intended composition and structural integrity over extended periods and throughout their lifecycle. Base alloys have a higher tendency to oxidate, which can alter their composition. Furthermore, the different melting points of the metals in base alloys can lead to uneven mixing and segregation during casting [15,16]. For example, chromium and nickel have significantly different melting temperatures [17], complicating the alloying process. From apractical standpoint, clinicians are unable to confirm the exact alloy composition of prosthetic materials.

With this in mind, the ADA established a classification system [18] for dental casting alloys that categorises alloys based on their composition. It divides alloys into three main groups:

1. High-noble alloys, characterised by a noblemetal content of a minimum of 60 wt% and a gold content of at least 40%.
2. Noble alloys, containing a noblemetal content of at least 25%, with no specific requirement for gold content.
3. Predominantly base metal alloys, possessing a noblemetal content below 25% [19].

The combination of thermomechanical, functional and biocompatibility properties define the clinical use of a dental alloy. The objective of this investigation was to analyse these properties of a novel high-noble Au–Pt–Ge porcelain-fused-to-metal alloy and determine its possible clinical applications for producing PFM dental restorations. Additionally, considering the need for an additional degassing procedure of the alloy to be used in the fabrication of metal–ceramic restorations, the null hypothesis stated that there is no statistically significant difference in cell viability when seeded on polished Au–Pt–Ge, oxidised Au–Pt–Ge and control samples.

## 2. Materials and Methods

### 2.1. Study Design for Determining Dental Alloy Properties for Clinical Use

**Figure 1:**
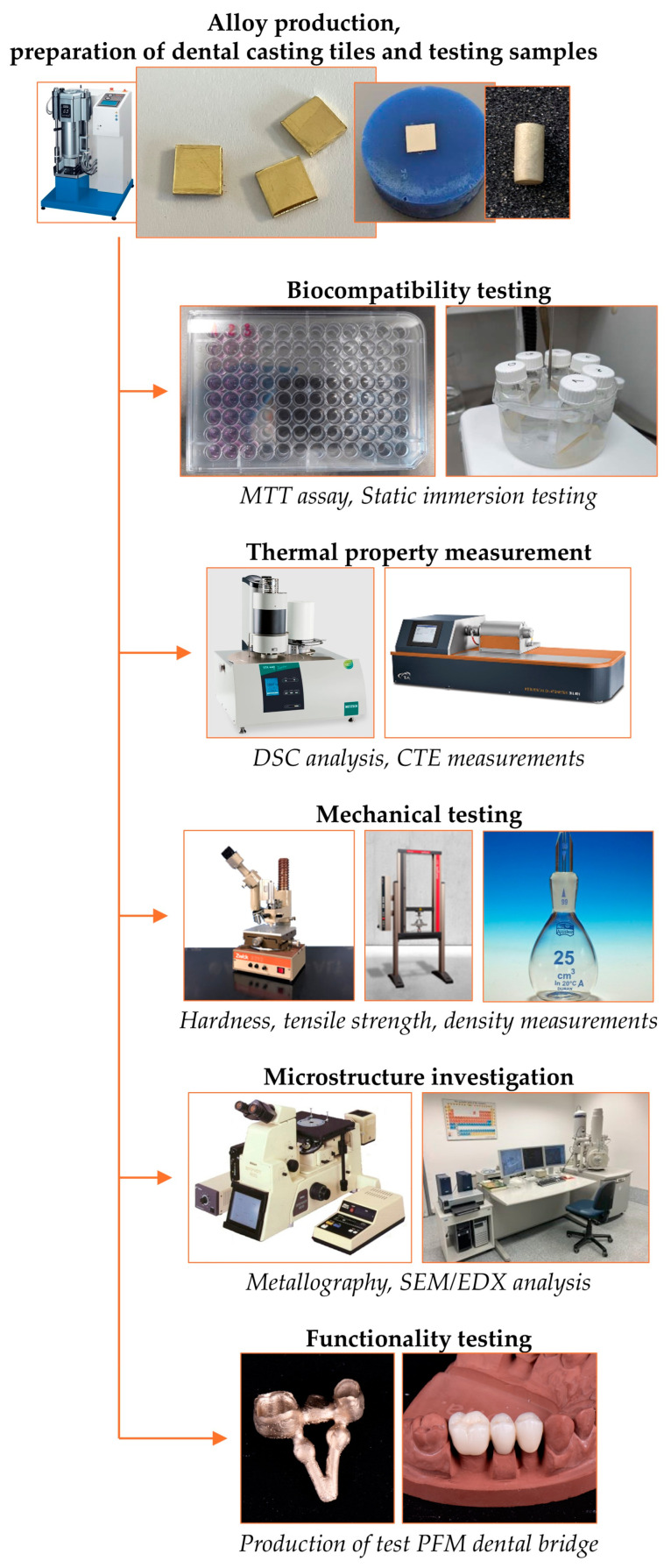
Performed investigations for determining the Au–Pt–Ge PFM dental alloy properties.

Information regarding biocompatibility, including thermal and mechanical properties and functional usefulness, is required for determining the suitability of a produced dental alloy for clinical use in PFM dental restorations. This investigation follows several types of analyses for this purpose, with regards to ISO 22674 for dental metallic materials for fixed and removable restorations [20]. The investigation of the developed dental alloy is shown in a diagram in Figure 1.

### 2.2. Alloy Production Experiment Parameters

The melting of pure components (Au = 99.99 wt.%, Pt = 99.99 wt.%, Ir = 99.99 wt.%, Ag = 99.99 wt.%, Ge = 99.99 wt.%, Rh = 99.99 wt.%) was performed in an induction vacuum pressure casting machine, the Galloni G3 (Aseg Galloni S.p.A., Milano, Italy). The initial melting parameters were at a vacuum of *p* = −0.6 bar and temperature of T = 1030 °C. At the onset of melting, the temperature was increased to 1120 °C and held for 2 min. The melt was then cast into a graphite crucible at a pressure of *p* = 0.6 bar, with an overpressure of *p* = 1.1 bar. The melt was held in the crucible for 6 min (360 s). The cast ingot had dimensions of 30 × 10 × 3 mm^3^.

After casting, the obtained alloy ingot was treated thermomechanically, using a procedure of profile rolling with intermittent thermal treatment. Rolling was performed from a flat profile of 3 × 10 mm^2^ with a thickness of 3 mm with three rolling steps with varying degrees of deformation and intermediate heating at a temperature of 700–750 °C for 15 min. This thermal treatment was performed in order to reduce the internal stress of the alloy caused by rolling, increase ductility through recrystallisation and enhance the homogeneity of the dental alloy microstructure. The material was rolled into a strip, reducing the thickness from 3 mm to 1.4 mm, followed by cutting into tile-shaped specimens with dimensions of 7 × 7 × 1.4 mm^3^.

The ternary system of the Au–Pt–Ge alloy had a nominal composition of 80Au-10.8Pt-1Ge and about 8 wt.% alloying elements (Ag, Ir, Rh).

### 2.3. Biocompatibility Evaluation

#### 2.3.1. Sample Preparation for Biocompatibility Testing

The samples used in the present investigation were divided into three groups (controlglass cover slip, oxidisedas-cast Au–Pt–Ge, and polished as-cast condition of Au–Pt–Ge).

The oxidised as-cast castings were cleaned initially by air blasting with 50 μm Al_2_O_3_ and then with 50 μm glass beads, followed by a 5 min ultrasonic cleaning in 95% ethanol. The degassing protocol included treatment in a ceramic furnace (Shofu dental laboratory, Songjiang, Shanghai, China). The heating was performed from 650 °C to 940 °C at a rate of 55 °C with a 5min hold at the peak temperature. The samples were then bench-cooled to room temperature. After degassing and heat treatment, the samples underwent additional air blasting with glass beads and ultrasonic cleaning in 95% ethanol.

The polished samples were prepared by polishing the air blasted samples with an abrasive wheel and rouge on a rag wheel, followed by a 10 min ultrasonic cleaning. These samples were also degassed and heat-treated. Following the heat treatment, they were polished and cleaned as before.

Before the biological research, the examined samples were sterilised in an autoclave for 15 min at 121 °C.

#### 2.3.2. MTT Assay

An in vitro primary biocompatibility test was conducted on primary human gingival fibroblasts (HGF), as previously described [21]. The gingival tissues were collected from healthy donors with written consent (approved by the institutional Ethical Committee (No. 36/7). The tissues were minced into 1 mm^3^ fragments and processed using the outgrowth method. These tissue fragments were then placed in 25 cm^2^ culture flasks containing a growth medium (DMEM/F12 with 10% FBS and 1% ABAM from Gibco, Thermo Fisher, Waltham, MA, USA) and incubated at 37 °C in a humidified 5% CO_2_ environment. The cells were passaged regularly when they reached 80% confluence, and the culture medium was changed every 3 days.

In accordance with ISO 10993-5:2009 [22], for the indirect test, the samples (control, Au–Pt–Ge and Au–Pt–Ge oxidised) were placed in a tube containing 10 mL of complete medium, incubated at 37 °C for two time intervals (1 and 7 days) and removed, while the remaining supernatants were used in further experiments. The cells were seeded in a 96-well plate (5000 cells/well), and the next day, 100 µL of supernatant was added to the corresponding wells (indirect MTT assay).

For the direct MTT test, the samples were placed onto 24-well plates; 20,000 cells/well were seeded onto discs and incubated in freshly prepared growth medium at 37 °C in a humidified 5% CO_2_ atmosphere for up to 7 days. The medium was changed every 3rd day. Mitochondrial activity was assessed after the 7th day of treatment.

For the assessment of mitochondrial activity after direct and indirect exposure to the tested samples, the medium was discarded, and 100 µL of solution containing 3-(4,5-dimethylthiazol-2-yl)-2,5 diphenyltetrazolium bromide (MTT, 0.5 mg/mL) (Sigma-Aldrich, St. Louis, MO, USA) was added to each well and incubated. After 4 h, the supernatant was discarded; dimethyl sulfoxide (Sigma-Aldrich, St. Louis, MO, USA) was added to each well, and the plate was placed on a shaker for 20 min at 250 rpm in the dark at 37 °C. The extracted coloured solutions from 24-well plates were transferred into a new 96-well plate. The optical density was measured at 550 nm using a microplate reader RT-2100c (Rayto, Shenzhen, China). The results were presented as a percentage of the control value.

### 2.4. Static Immersion Testing

#### 2.4.1. Preparation of Solution—Artificial Saliva

A solution of artificial saliva was prepared according to the ISO 10271 Standard [23]. First, 5850 g of NaCl and 10.0 g of >85% C3H6O3 were dissolved in 300 mL of water. Distilled water was then added to the solution to a total volume of 1000 mL. The prepared solution had a pH value of 2.24, meeting the Standard’s requirements.

#### 2.4.2. Sample Preparation

Two samples for static immersion testing were prepared in accordance with ISO 10271, with the dimensions 34 × 13 × 1.5 mm^3^ and a total surface area of 10.25 cm^2^. Prior to immersion testing, the samples were cleaned in an ultrasonic bath with ethanol, rinsed with distilled water and dried with dry air. After cleaning, they were measured and weighed.

#### 2.4.3. Testing Procedure

The samples were immersed in 20 mL of each test solution in a test tube closed with a rubber cork using nylon string. The test duration was 168 h (7 days) at a constant temperature of 37 ± 0.2 °C. Parallel with the samples, a reference pure solution with no samples was treated in the same way. To evaluate the migration of ions from the samples into the solution, the chemical content of the suspensions was overseen by ICP-MS,( HP, Agilent 7500 CE, equipped with a collision cell (Santa Clara, CA, USA)).

### 2.5. DSC Analysis

DSC analysis of the obtained dental alloy was conducted to determine the onset of melting enthalpy. The sample for measurement had dimensions of approximately 2 × 2 × 2 mm^3^ and weighed 40.63 mg, taken from the thermomechanical treatment of the alloy.

For determining the melting interval, we performed an additional analysis using a sample weighing 0.1633 g. The analysis programme involved heating at 10 K/min up to a temperature of 1300 °C and cooling to room temperature at 20 K/min. The analysis was carried out in an argon protective atmosphere. The analysis was performed using an NETZSCH STA 449 F3Jupiter device (Netzsch-Gerätebau GmbH, Selb, Germany).

### 2.6. Dilatometric Coefficient (CTE) Analysis

Testing of the thermal expansion coefficient (CTE) was conducted on a DIL 805A/D mechanical dilatometry device (TA Instruments, New Castle, DE, USA). The measurements were performed on 3 samples (dimensions Φ4 ×10 mm). The results are presented in a line graph.

### 2.7. Hardness

According to the Standard 6507-1:1997 [24], the measurements of hardness were conducted using the static Vickers test on the Zwick 3212 microhardness measurement device (ZwickRoell AG, Ulm, Germany). An applied load of F = 49 N was used for testing the samples, as per the Standard. Six measurements were performed for each sample.

### 2.8. Tensile Testing

**Figure 2:**
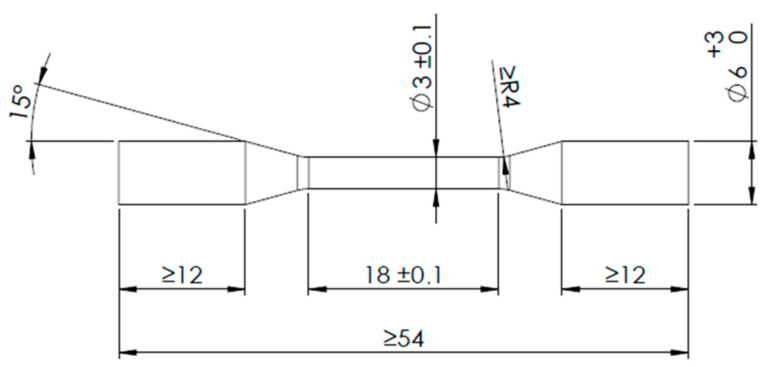
Specimen dimensions for tensile testing of mechanical properties(unit: mm).

Tensile testing was performed on a ZWICK/ROELL Z010 (Zwick Roell Group, Ulm, Germany) material testing machine. Four samples of the produced alloy were prepared for testing, with shapes and dimensions according to ISO 22674 [20]. The test specimens had two conical shoulders, with dimensions shown in Figure 2. The preload was 0.1 MPa, and the test speed was 2 mm/min. The tests were performed to determine the 0.2% proof stress, elastic modulus, tensile strength and elongation at break.

### 2.9. Density Measurements

The density was measured with a 25 mL pycnometer and deionised water. The pycnometer was first weighed empty. It was then weighed with the cast alloy sample. This was followed by five measurements of the water-filled pycnometer without a sample and five measurements of the water-filled pycnometer with the cast alloy sample. A scale with four decimal places was used for weighing. The temperature of the water was 19 °C, and the density of the water was 0.998 g/cm^3^. The density of the cast sample was calculated from the five measurements of the pycnometer displaced water volume and known weight of the sample.

The measured density was calculated from the pycnometer weight measurements using the equation $\rho_{sample}=\frac{m_{sample}\cdot \rho_{liquid}}{m_{pycnometer+liquid}\;+\;m_{sample}\;-\;m_{pycnometer+liquid+sample}}$ and a measured mass of 3.03 g for the alloy sample. The measured density was used for the production of a test dental bridge with the lost-wax cast method.

### 2.10. Microstructure Investigations

The produced alloy casting tile sample for the microstructure investigations was cold mounted and ground with 600 and 1200 grit size. This was followed by polishing with 1 μm and 0.05 μm aluminium oxide suspensions. After polishing, etching was performed using a solution prepared by mixing 30 mL distilled water, 25 mL HCl and 5 mL HNO_3_. The sample was etched for 60 s and then rinsed with ammonium hydroxide and distilled water. A brief manual polishing with 0.05 μm aluminium oxide suspension was performed, followed by cleaning with distilled water in an ultrasonic bath.

The microstructure of the finally polished and etched sample was investigated with an optical metallographic microscope, Nikon Epiphot 300 (Tokyo, Japan), equipped with a CCD camera Olympus DP12 (Boston, MA, USA). A grain size analysis of the alloy was performed with the planimetric method according to ASTM E112. A scanning electron microscope (SEM), Sirion 400NC (FEI, Hillsboro, OR, USA) was used for detailed microstructure observation with an INCA 350 (Oxford Instruments, Abingdon, UK) energy-dispersive *X*-ray spectroscopy detector for microchemical analysis.

### 2.11. Production of a Test PFM Dental Bridge

A 3-unit dental bridge substructure was modelled by hand using wax. The refractory material used was phosphate-based Interfine K+B Speed (Interdent d.o.o., Celje, Slovenia). The refractory mass was mixed according to the manufacturer’s instructions for precious alloys with Expasol liquid (Interdent d.o.o., Celje, Slovenia) and distilled water, with a ratio of 60:40. The rapid heating method was used for the refractory block. After 20 min of mixing the powder and liquid of the refractory mass, the cuvette was introduced into an annealing furnace preheated to 850 °C. After holding for 40 min at this temperature, casting was started in a furnace with an induction heater. A graphite crucible was used to melt the alloy. The alloy melted completely very quickly (in a few seconds) and was poured into a heated refractory block using a centrifuge. The refractory block was cooled spontaneously to room temperature. The cast substructure was removed from the refractory block.

The opaquers and faceted ceramics used were manufactured by IvoclarVivadent AG (Schaan, Liechtenstein). After processing, a wash opaquer was applied and fired at 890 °C. After the wash layer, two more opaquer layers were applied, fired at 870 °C. The IPS Style Ceram Powder Opaquer was used.

After the opaquer, an IPS Style Ceram Dentin was applied and fired at 800 °C, according to the Standard programme for the given ceramic in the Ivoclar ceramic oven. The dentin layer was followed by a second firing, the incisal layer, using IPS Style Incisal, also at 800 °C. The faceted ceramic used was in the A1 colour, glazed at 770 °C.

The thin edge of the crowns, which was not covered by ceramic, was polished after glazing with a green polishing stone for metal polishing and a yellow polishing stone for polishing ceramics. A high gloss and smoothness of the alloy were obtained.

### 2.12. Statistical Analysis

The data were analysed using IBM SPSS Statistics v22 software (SPSS Inc., Chicago, IL, USA). Hardness, tensile testing and density measurements were reported using descriptive statistics, expressed as the mean and median with standard deviation (SD). The results of the coefficient of thermal expansion (CTE) analysis were presented in a line graph.

A one-way analysis of variance (ANOVA) test was used for evaluating the differences between both the Au–Pt–Ge samples (Au–Pt–Ge in as-cast oxidised and polished forms) and the control. A *p*-value less than 0.05 was considered statistically significant. The sample size for this study was determined based on the primary variables, including cell viability, as key outcome. Using G*Power 3.1.9.7 software, with a significance level (α) of 0.05, a power (1 − β) of 0.80, and an estimated effect size 0.85, the minimum required sample size was calculated to be 18 for all experimental groups (indirect 24 h, indirect 7 days and direct 7 days).

## 3. Results

### 3.1. Biocompatibility

**Figure 3:**
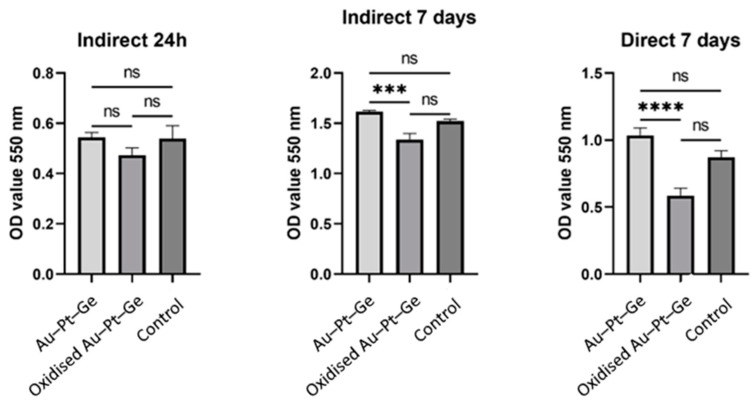
Optical density measurements for the MTT assay of cell viability (statistical significance level: ns—not significant; ***—very significant; ****—extremely significant).

After 24 h of indirect exposure of human gingival cells (HGCs) to the tested materials, the mitochondrial activity observed in the tested groups (control and polished Au–Pt–Ge) was almost similar. Lower activity was observed in cells treated with oxidised Au–Pt–Gesupernatants, but there were no statistically significant differences compared to the control and polished Au–Pt–Ge samples (Figure 3).

After 7 days of both indirect and direct exposure, it was observed that cell viability increased in contact with the as-cast Au–Pt–Ge and decreased in contact with the oxidised supernatants compared to the control. When comparing the cells’ mitochondrial activity between the groups (the control group, the cells seeded on Au–Pt–Ge and the cells seeded on the oxidised Au–Pt–Ge samples), the ANOVA test indicated statistically significant differences only between the oxidised and Au–Pt–Ge groups in terms of both indirect and direct contact (Figure 3).

### 3.2. ICP Analysis of Solutions After Immersion Testing

As seen from Table 1, the total ions released for the Au–Pt–Ge dental alloy were 1622 μg/cm^2^ per 7 days. According to ISO 22674 [20], the total metal ion release should not exceed 200 μg/cm^2^ in a time period of 7 days ± 1 h. The measured metal ion release was well below this limit.

### 3.3. DSC Results

**Figure 4:**
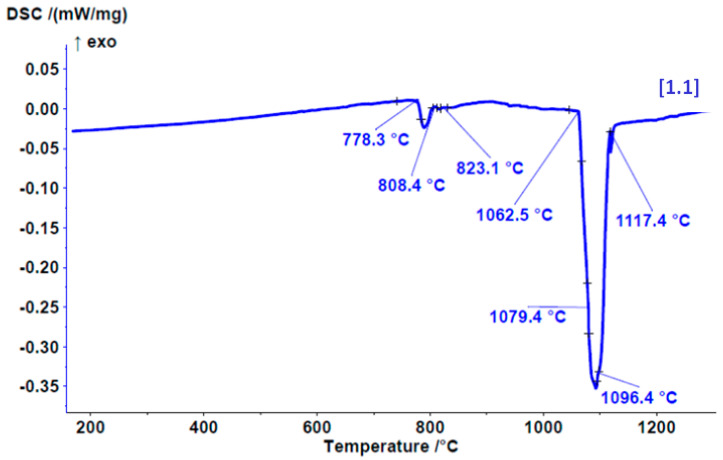
DSC heating curve for the Au–Pt–Ge alloy (+ − tangent line intersections with curve, tangent lines not shown).

The onset of melting enthalpy, or the melting point, was found to occurat 1069.71 ± 3 °C. The DSC heating curve (Figure 4) indicates the melting of a dental alloy, starting at 778 °C, where a low-melting eutectic is likely to melt. Within this temperature range, three different phases/eutectics also undergo melting. The main melting portion begins at 1062 °C in three steps or phases.

**Figure 5:**
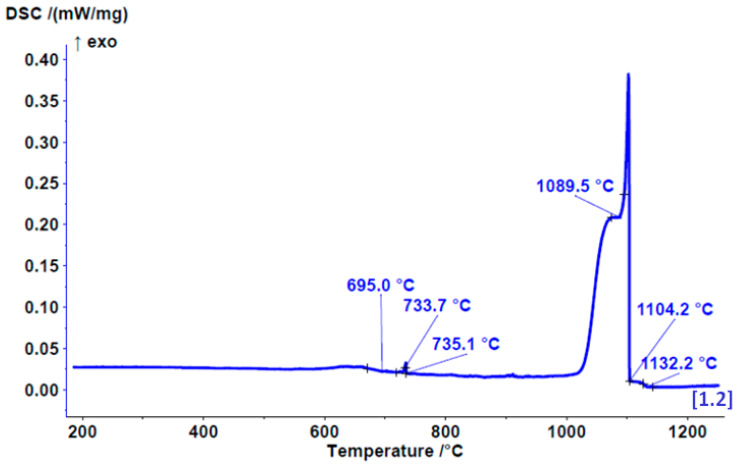
DSC cooling curve for the Au–Pt–Ge alloy (+ − tangent line intersections with curve, tangent lines not shown).

The start of solidification is revealed by the cooling DSC curve at 1132 °C with three solidification temperatures/phases. Similarly, on the cooling curve (Figure 5), the solidification of three different phases/eutectics can be observed within a range of approximately 700 °C. This leads to the conclusion that six different phases appear in the microstructure.

### 3.4. CTE Results

**Figure 6:**
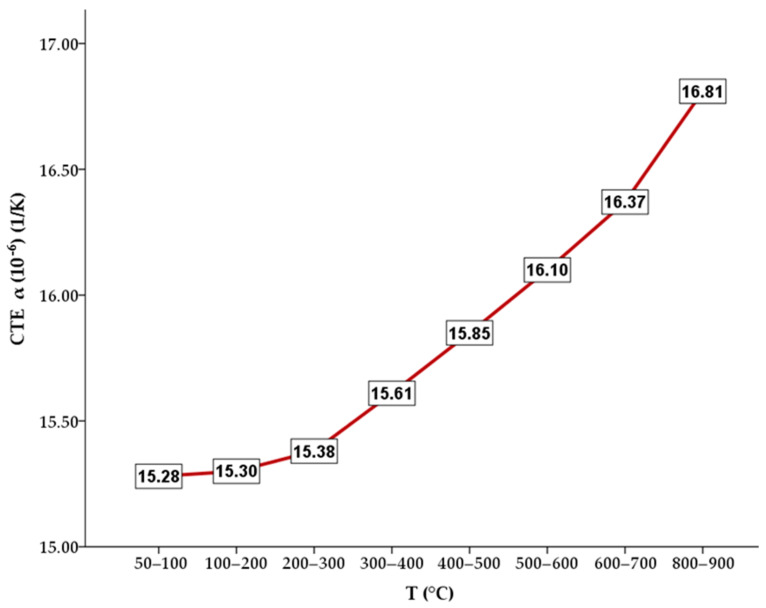
CTE measurement results for the Au–Pt–Ge alloy.

Testing of the thermal expansion coefficient showed that the average value of CTE (50–900 °C) for the Au–Pt–Ge alloy was about 15.84 × 10^−6^ K^−1^ (Figure 6). The average CTE value for the range of 50–500 °C was 15.48 × 10^−6^ K^−1^. The CTE values for conventional high-noble dental alloys for PFM restorations are about 14 to 15 × 10^−6^ K^−1^ (based on a general overview of different noble metal dental alloys from manufacturers) with coefficients of dental alloys for PFM restorations generally being about 12.7–14.8 × 10^−6^ K^−1^ [25,26].

### 3.5. Microhardness

The average results of the hardness measurement are presented in Table 2. As shown, the average microhardness value was 127.17 HV. The average hardness is somewhat lower than typical values for high-noble dental alloys for PFM restorations, which ordinarily have a hardness about 170 HV.

### 3.6. Tensile Testing Results

**Figure 7:**
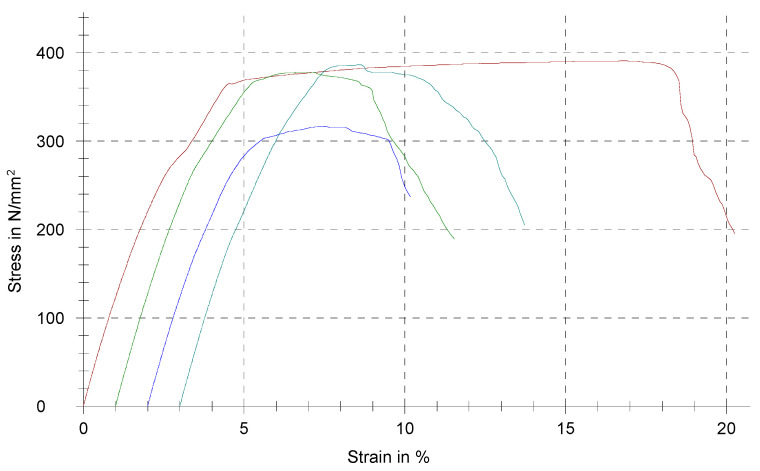
Chart of tensile testing results of the Au–Pt–Ge alloy (each of the four samples displayed with a different colour. Red line: sample No1, green line: sample No2, dark blue line: sample No3, light blue line: sample No4).

The tensile testing results are shown in the chart in Figure 7, with 0.2% proof stress (Rp 0.2), gauge length (L0), elastic modulus (E-Modulus), tensile strength (Rm) and elongation at break (e Break) given in Table 3. According to ISO 22674 [20], the alloy belongs to type III dental alloys, with a minimum proof stress of 270 MPa and minimum elongation after fracture of 5%. No criteria for a minimum elastic modulus are given for these types of dental alloys in the standard.

### 3.7. Density

The average measured density of the alloy sample was 16.46 ± 0.23 g/cm^3^, as shown in Table 4. The measured density of the alloy is within typical values for high-noble metal dental alloys.

### 3.8. Microstructure Investigation Results

**Figure 8:**
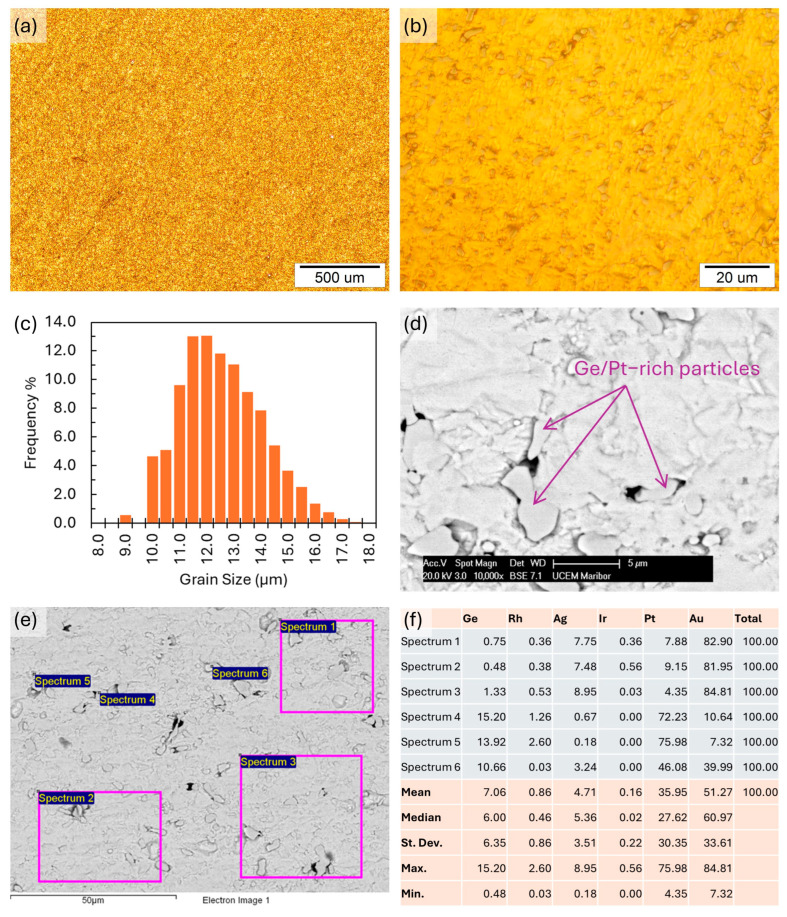
Microstructural investigations of the cast alloy. (**a**) Optical image of the microstructure at 10× magnification. (**b**) Optical image of the microstructure at 100× magnification. (**c**) Grain size distribution from the ASTM grain size analysis. (**d**) SEM image of the microstructure at 10,000× magnification. (**e**) SEM image with locations for EDX analysis. (**f**) EDX analysis results.

The microstructural investigations are presented in Figure 8. The optical metallography in Figure 8a,b shows a homogeneous microstructure. The ASTM grain size analysis result for the alloy grain size number G was 13.16, with a mean intercept distance of 3.35 µm. The frequency of the grain size distribution is shown in Figure 8c. The SEM images show a similar homogeneous microstructure. The EDX microchemical analysis shows an alloy matrix with the initial basic composition, along with Ge- and Pt-rich particles with sizes of about a few μm up to around 10 μm. Examination of the Ge/Pt-rich particles in the matrix showed a relatively homogeneous distribution, with no visible areas of particle clustering or larger particles present, which would indicate areas of higher Ge concentration in the matrix. Figure 8f shows the results of an EDX analysis of the matrix and Ge/Pt-rich particles typical for the sample surface shown in Figure 8d,f.

### 3.9. Produced Test PFM Dental Bridge

**Figure 9:**
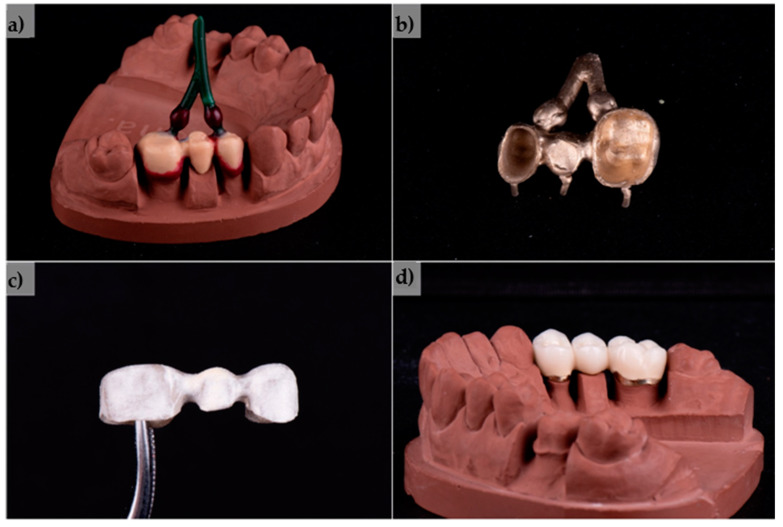
Some highlighted steps of the test PFM dental bridge production, with the initial wax model (**a**), alloy casting with sprues (**b**), opaquer firing (**c**) and the appearance of the finished product (**d**).

The produced three-unit dental bridge casting was completely homogeneous and compact, with a nice golden colour. The processing of the metal substructure was not difficult, without the need for great force or a high number of revolutions of the cutter. Moderate heating of the metal during processing was observed, which was expected due to friction. The overall impression is that machining is faster and easier than for CoCr alloys. A high gloss and smoothness of the alloy were obtained with polishing and glazing, which were also faster and easier than with base metal alloys. The finished product had a noticeably pleasant colour and excellent optical characteristics of the ceramics on the given alloy (Figure 9).

## 4. Discussion

This study was aimed primarily to investigate the biomechanical properties of the novel ternary noble Au–Pt–Ge alloy and its possible clinical applications for producing PFM dental restorations. For the development of a dental alloy, we chose the composition described above (80% Au; 10.84% Pt; 1% Ge; 8% alloying elements). Historically, the main noble alloys utilised in dental prosthetics contained significant amounts of palladium. Recent research has shown that palladium can cause allergic reactions, even though it is considered a noble metal [27,28], although the incidence of palladium allergies is considered relatively rare and occurs in connection with nickel allergies [29]. This has led to the development of a new palladium-free alloy made of gold, platinum and germanium to reduce the potential adverse effects associated with palladium [30,31]. Additionally, a higher Pt content is essential to elevate the melting range effectively beyond the porcelain firing temperature, thereby preventing distortion during the porcelain application phase. According to the firing temperature, dental porcelains are divided into four categories: ultra-low fusing (≤850 °C), low fusing (850–1100 °C), medium fusing (1100–1300 °C) and high fusing (≥1300 °C) (the latter of which are used less frequently) [32]. So, for dental alloys used in porcelain-fused-to-metal (PFM) restorations, the key physical requirement is a melting point of nearly 1100 °C. However, most of the alloys with high Au content that meet the Standards’ requirements for PFM application melt at temperatures below 1000 °C, which makes them easier to cast than base alloys. However, gold alloys designed for porcelain-fused applications must have a solidus temperature at least 100 °C higher than the firing temperature of dental porcelain, which, for most veneering porcelains, is between 960 and 980 °C. This higher temperature is crucial to ensure that the frameworks maintain their shape and do not sag during firing [33]. Based on the results obtained from DSC analysis, the melting of the novel Au–Pt–Ge alloy begins at 1069.71 ± 3 °C, making it suitable for low-fusing dental porcelains. Additional modifications to the alloy composition would be required to increase its melting point for usability with medium-fusing dental porcelains.

Considering further thermomechanical features, high-noble alloys are produced carefully to have a coefficient of thermal expansion (CTE) that matches that of dental porcelain closely. The metal alloy is typically designed to have a slightly higher CTE than the porcelain, with the coefficients generally falling within the intervals of 12.7–14.8 × 10^−6^ K^−1^ for the alloys and 10.8–14.6 × 10^−6^ K^−1^ for the porcelain [25,26]. This slight mismatch in CTE values serves a specific purpose. As the materials cool from the high firing temperatures used during the porcelain application process, the metal contracts slightly more than the porcelain. This differential contraction places the porcelain in a state of slight compressive stress. Such compressive stress is beneficial, as it helps prevent the development of tensile stress, which could lead to cracking or debonding of the porcelain from the metal substructure. The induced compressive stress enhances the bond’s strength and stability, minimising the risk of mechanical failure under the functional loads experienced in the oral environment. By ensuring that the CTE values of the alloy and porcelain are well-matched yet strategically different, high-noble alloys provide a stable base for durable and reliable dental restorations [7]. In cases where the difference in CTE between the alloy and porcelain is greater than 1 × 10^–6^ K^−1^, increased residual stresses occur, resulting in the delamination of the porcelain from the metal substrate [34]. The CTE results show an average CTE value of 15.48 × 10^−6^ K^−1^ for the heating range of 50–500 °C. The alloy is applicable for use with porcelains that are compatible, such as the one used in this study with a CTE alloy range of 13.8–15.2 × 10^−6^ K^−1^ (25–500 °C) (CTE information available by the manufacturer, IvoclarVivadent AG).

Considering the results of the sample hardness measurements, it was observed that the alloy demonstrated lower hardness but still met the minimum values required for dental applications in fixed restorations, including up to three-unit bridges [35]. For single crowns and up to three-unit bridges, the hardness of the selected alloy should be at least 130 HV [33]. The tested samples, irrespective of the fabrication method, are not suitable for multiunit fixed prostheses, especially for extensive multiunit fixed restorations. The metallographic investigation showed a homogeneous, finely grained microstructure, with a grain size number G of 13.16. The microstructure and chemical analyses showed apparent Ge segregation. Ge segregation decreases the alloy’s mechanical properties and may affect workability negatively, as the alloy becomes more brittle with higher additions of Ge [36]. To reduce these negative effects, the Ge distribution must be homogeneous in the matrix. The observations and analyses of the microstructure showed an even concentration of Ge across the sample, indicating that a consistent distribution had been achieved.

The tensile testing results showed similar findings regarding the clinical use to those of the hardness measurements. The alloy achieved a minimum proof stress of 270 MPa and minimum elongation after fracture of 5%, which categorises it in the type III dental alloys, according to ISO 22674 [20]. Type III dental alloys are applicable for multiple-unit fixed prostheses but are not available for appliances with thin sections that are subject to very high forces, such as full-arch fixed dental prostheses and implant-retained superstructures.

Additional density measurements of the alloy were performed to facilitate the production of the test PFM dental bridge. The measured density showed typical values for high-noble dental alloys suitable for dental restorations of this type. The production of the dental bridge was performed with no major difficulties or defects in the casting. The substructure was homogeneous, while the processing and machining were less demanding and faster than with base metal or typical CoCr alloys. The ceramic firing also did not introduce any defects or sagging of the metallic substructure. The appearance of the finished PFM dental bridge was of a high standard for dental restorations, with excellent optical characteristics of the ceramics.

The metal ion release test demonstrated that the alloy is biologically safe for use in the oral environment. According to the ISO 22674 Standard for metallic dental materials [20], it is required that the cumulative metal ion concentration released from the alloy should not exceed 200 μg/cm^2^ within a 7-day period ± 1 h at a temperature of 37 ± 1 °C. The Au–Pt–Ge dental alloy complies with these criteria, as the measured ion release is minimal, making it suitable for application in dental prosthetics. No noble metal ions were detected from the ion release, while small amounts of released Ge were detected. As the investigated alloy is based on noble metals, it has a high corrosion resistance, and the measured metal ion release is well below the values of ion release in conventional base metal alloys measured in similar investigations [37,38]. In these investigations, the total metal ion release can be significantly higher, up to 20 μg/cm^2^ within a 7-day period [37]. Additionally, Ge is considered a trace component in the human diet, with little information on the toxicity of inorganic Ge compounds to humans [39].

The biocompatibility assessment of noble alloys has been extensively investigated through multiple in vivo and in vitro studies [40,41,42,43]. The primary emphasis in these investigations has centred on assessing the performance of polished porcelain-fused-to-metal (PFM) alloys. Overall, these studies conclude that these alloys are generally safe and non-toxic when polished [43].

Our study examined cell viability following plating for the degassing as-cast condition and polished samples. The MTT test was selected as the primary biocompatibility test, as it assesses mitochondrial activity, which may be compromised due to the effect of metals on cellular oxidative stress. We analysed mitochondrial activity after 24 h and 7 days, since previous investigations [24] highlighted those time frames as significant periods when differences are noticeable. Also, corrosion research standards require seven days of immersion testing, so we intended for all investigations to be uniform. Based on the obtained results, the null hypothesis was rejected. Significantly higher viability ratings were noted on the polished samples. This result is supported by a similar study conducted on an Au-based PFM alloy, which demonstrated the improved biocompatibility of polished casting alloys compared to their as-cast counterparts after the degassing process [44,45,46,47]. Cells in contact with the polished samples had higher optical densities than those in contact with the samples after degassing treatment [40,48]. Oxidised forms induced changes in cells, including actin filament disintegration, indicating a mild to moderate response [48].

Additionally, reactions in gingival tissues near dental alloys are more frequent than previously thought, with chronic inflammation and metal ion accumulation observed. While adding non-noble elements like In, Sn or Zn improves porcelain adhesion, it also increases corrosion and metal ion release, raising toxicity concerns [44,45,46,47]. High-noble alloys generally demonstrate better biocompatibility, though heat treatment heightens ion release and reduced cell viability, especially if the alloying element is zinc [49,50]. To minimise ion release and protect oral health, removing the oxide layer is recommended.

Although a significant portion of the metallic substructure is covered by veneering porcelain, typically, the cervical part, i.e., the collar, remains exposed. As it is situated subgingivally, thereby in direct contact with the surrounding tissue and susceptible to exposure to oral and subgingival fluids, this area needs to be highly polished.

Based on the discussed analyses of the manufactured alloy, its thermomechanical properties are suitable for clinical applications in dentistry for prosthetic products as a type III alloy. It may not be used for extensive multiunit fixed restorations, as the Ge segregations in its microstructure reduce its mechanical properties. The alloy shows excellent workability for the dental technician but a relatively low melting point and slightly higher CTE, requiring attentive selection of processing parameters and porcelain combinations for the production of PFM dental restorations with a high standard for clinical use. From metal ion release and biocompatibility assessments, it may be concluded that the Au–Pt–Ge alloy is highly suitable for patients prone to metal allergies.

While we compared the obtained results with standards for testing materials intended for use in dental prosthetics, a limitation of this study is the lack of a control alloy as a reference point for comparison. This may create challenges in determining whether the observed properties are due to the alloy composition, the processing methods employed or other external factors, despite adhering to established standards.

## 5. Conclusions

Based on the conducted investigation on the development of an Au–Pt–Ge based dental alloy, the following conclusions may be derived:

- Based on the analyses of the manufactured alloy, its thermomechanical properties are suitable for applications in dentistry for prosthetic products as a type III alloy. It is suitable for multiple-unit fixed prostheses, while not being appropriate for extensive multiunit fixed restorations. The cast alloy has a finely grained microstructure with Ge segregations, which decrease its mechanical properties.
- A relatively low melting point and slightly higher CTE than other conventional dental alloys requires the careful selection of processing parameters and porcelain combinations for the production of PFM dental restorations.
- The measured metal ion release from immersion testing was minimal, demonstrating that the alloy is biologically safe for use in an oral environment and is highly suitable for patients prone to metal allergies.
- The production of the three-unit PFM dental bridge was performed with no major difficulties in processing, being less demanding and faster than with base metals and resulting in the production of a high-quality dental restoration.
- The cell viability examination for as-cast and polished alloy samples showed significantly higher viability ratings on the polished samples, demonstrating the improved biocompatibility of polished casting alloys compared to their as-cast counterparts. The results show that a dental substructure in direct contact with the surrounding tissue and susceptible to exposure to oral and subgingival fluids should be highly polished.

## Figures and Tables

**Table 1:** Results of the ICP analysis in µg/cm^2^ after 7 days of immersion of the Au–Pt–Ge sample in artificial saliva (pH 2.24).

Ag	Au	Be	Cd	Ge	In	Ir	Ni	Pb	Pd	Pt	Rh	Total
0	0	0	0	1.622	0	0	0	0	0	0	0	1.622

**Table 2:** Results of the microhardness measurements.

Measurement	Microhardness HV5
1	130.1
2	125.4
3	129.6
4	128.1
5	124.9
6	124.9
Mean	127.17
Median	126.75
Min.	124.9
Max.	130.1
St. Dev.	2.19

**Table 3:** Tensile testing results of the Au–Pt–Ge alloy.

Measurement	Rp 0.2 (N/mm^2^)	L0 (mm)	E-Modulus (N/mm^2^)	Rm (N/mm^2^)	e Break (%)
1	266.12	15.00	10,846.07	390.39	20.26
2	277.65	15.00	11,397.78	377.50	10.53
3	266.50	15.00	10,665.14	316.24	8.17
4	286.68	15.00	10,931.50	386.20	10.72
Mean	274.24	15.00	10,960.12	367.58	12.42
Median	272.08	15.00	10,888.79	381.85	10.63
Standard Dev.	8.55	0.00	270.36	30.01	4.64

**Table 4:** Density measurements of the Au–Pt–Ge alloy.

Measurement	Mass of Pycnometer and Liquid Without Sample (g)	Mass of Pycnometer and Liquid with Sample (g)	Alloy Density (g/cm^3^)
1	40.4202	43.2638	16.1659
2	40.4197	43.2659	16.3937
3	40.4171	43.2676	16.7849
4	40.4173	43.2640	16.4383
5	40.4182	43.2661	16.5462
Mean	40.4185	43.2655	16.4633
Median	40.4182	43.2659	16.4383
Standard Dev.	0.0014	0.0016	0.2259

## Data Availability

The data presented in this study are available on request from the corresponding author.
